# Intermittent stuck valve after aortic valve replacement with a mechanical valve: A case report

**DOI:** 10.1097/MD.0000000000006214

**Published:** 2017-03-03

**Authors:** Wenzong Luo, Xinxin Wang, Jing Li, Yun Mu, Yiming Ni

**Affiliations:** a Department of Thoracic and Cardiovascular Surgery, First Affiliated Hospital of Zhejiang University, School of Medicine, Hangzhou; b Department of Thoracic and Cardiovascular Surgery, Affiliated Taizhou Hospital of Wenzhou Medical University, Taizhou; c Department of Ultrasound, First Affiliated Hospital of Zhejiang University, School of Medicine, Hangzhou, China.

**Keywords:** aortic valve replacement, case report, stuck valve

## Abstract

**Background:**

Intermittent stuck valve after mechanical valve replacement surgery is a very rare and severe complication.

**Case summary:**

We present 1 case of a 53-year-old woman after aortic valve replacement for severe aortic valve stenosis combined with hypertrophy septum. She was diagnosed with intermittent stuck valve only 1 day after surgery by clinical symptoms, intraoperative transoesophageal echocardiogram, and intraoperative findings.

**Conclusions:**

Although indications for concomitant myectomy during aortic valve replacement are not clear, we recommend myectomy to prevent stuck valve after St Jude Medical Regent prosthesis replacement for severe aortic valve stenosis combined with hypertrophy septum.

## Abstract 2

Supplemental Digital Content is available in the text

## 1 Introduction

By improvement of prosthetic valve and application of intraoperative transoesophageal echocardiogram (TOE), the operative results of valve replacement became generally satisfactory. Moreover, with a better understanding of the mechanism of valve disease such as aortic stenosis combined with hypertrophic cardiomyopathy, safe and effective surgical procedures were developed. Stuck valve immediately after valve replacement was prevented with intraoperative TOE. However, here we present a case of delayed intermittent stuck valve after surgery.

## 2 Case report

**Figure 1:**
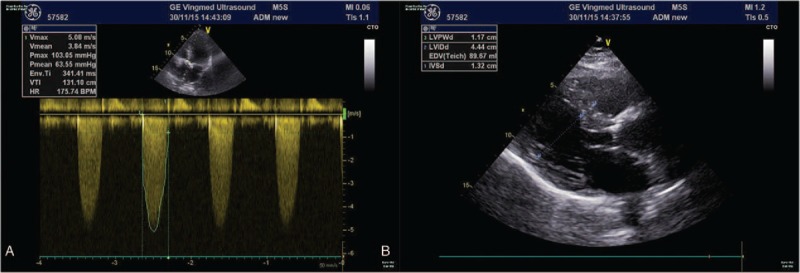
(A) The transthoracic echocardiography before surgery. The echocardiography shows a peak pressure gradient of 103 mmHg. (B) The transthoracic echocardiography before surgery. The echocardiography shows a septum thickness of 1.32 cm.

**Figure 2:**
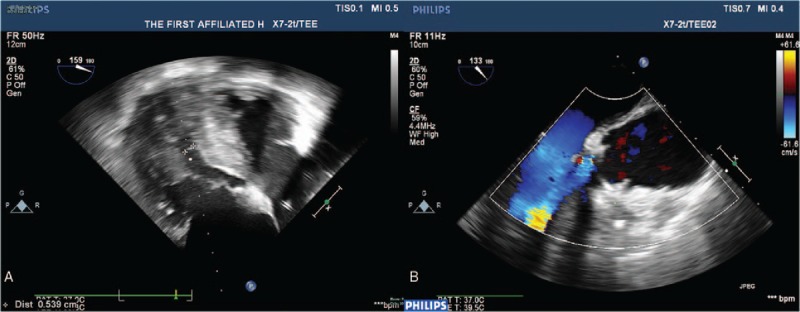
(A) The intraoperative transoesophageal echocardiogram before second surgery. There was a severe aortic regurgitation. (B) The intraoperative transoesophageal echocardiogram before second surgery. The left ventricle outflow tract was as narrow as 0.539 cm owing to the bulged septum.

**Figure 3:**
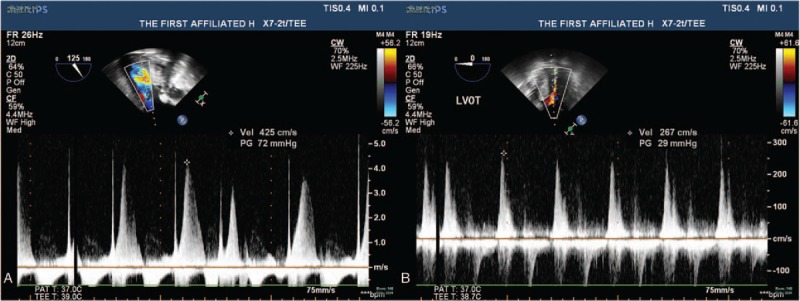
(A) The intraoperative transoesophageal echocardiogram before second surgery. The outflow tract flow velocity was 4.3 m/s. (B) The intraoperative transoesophageal echocardiogram after second surgery. The transvalvular flow velocity dropped to 2.7 m/s after the second surgery.

A 53-year-old woman was admitted because of shortness of breath and impaired physical capacity (New York Heart Association class II-III). On preoperative echocardiogram, severe congenital bicuspid aortic valve stenosis was confirmed with a peak transvalvular pressure gradient of 103 mmHg and septum thickness of 1.32 cm (Figure 1A and 1B). Calcified stenotic valve did not work well on echocardiogram. The aortic valve was replaced with a 19-mm St Jude Medical Regent prosthesis (St Jude Medical, Inc, St Paul, MN). The cuff of the valve prosthesis was raised above the tissue annulus, whereas the struts remained intra-annular. One of the pivot guards was placed in contact with interventricular septum. Normal leaflet mobility and pressure gradient without systolic anterior motion (SAM) were confirmed on TOE. Hemodynamics became unstable on the second day and with a large dose of vasoactive drugs the systolic pressure was held just >100 mmHg. No acute hemorrhage was observed according to drainage. A fast transvalvular flow velocity of 2.3 m/s was revealed by the transthoracic echocardiogram (TTE). The situation became worse on the third day as the systolic pressure sometimes jumped to 220 mmHg or dropped to 75 mmHg. The dose of vasoactive drugs was adjusted frequently according to the changeable blood pressure. The continuous cardiac output monitor showed intermittent rises and drops of cardiac output. A faster transvalular velocity of 4.5 m/s and peak pressure gradient of 79 mmHg were revealed on TTE. An emergent surgery was performed. The intraoperative TOE showed that the basal septum bulged into the left ventricle outflow tract (LVOT) with a thickness of 1.6 cm increasing by about 20% from baseline and the leaflets of the prosthesis did not work well accompanied with severe aortic regurgitation and a fast outflow tract flow velocity (Fig. 2A, 2B, 3A, Supplemental Video). In surgery, we observed that the basal septum bulged into the LVOT and interfered the mobility of both leaflets. After myectomy and replacement with a new 19-mm St Jude Medical Regent prosthesis, valve worked well on intraoperative TOE (Fig. 3B). However, the blood pressure was not stable to wean cardiopulmonary bypass. Although the patient was supported with extracorporeal membrane oxygenation, the pressure and O_2_ saturation dropped still. The patient was declared death finally.

## 3 Discussion

St Jude Medical Regent prosthesis is designed to have low profile structure and unique pivot systems. In the aortic position, one of the pivot guards of a St Jude Medical Regent prosthesis placed in the native anulus often comes into contact with left ventricular wall or the interventricular septum in either valve orientation.^[1,2]^ In our case, the hypertrophy septum increases the risk of stuck valve for a St Jude Medical Regent prosthesis in aortic position.

Intermittent stuck valve is a rare complication after valve replacement, which is mostly caused by thrombus or pannus formed on valve because of ineffective anticoagulation. Clinical symptoms were varied from dyspnea at rest, low output, chest pain, shock, effort intolerance, embolization, cardiac arrest to asymptom according to the type and location of prosthesis.^[3]^ Echocardiogram is a key examination to diagnose stuck valve. However, in our case, stuck valve was not observed on TTE before second surgery except for a fast transvalvular velocity. The most significant indication for stuck valve was the “roller coaster” like blood pressure. During the second surgery, the intraoperative TOE revealed that the valve working abnormally accompanied by a severe aortic regurgitation providing reliable evidence for stuck valve since we ruled out the possibility of periprosthetic leak in surgery. Also we found the thickened septum bulged into LVOT and contacted the pivot of the valve severely influencing the valve movement. After myectomy and replacement with a same size St Jude Medical Regent prosthesis, the valve worked well on TOE. Based on it we are convinced that the thickened septum was one of the key factors for stuck valve.

Cardiac hypertrophy involving the septum is one characteristic of severe aortic valve stenosis.^[4]^ Panza and Maron^[4]^ have recommended concomitant myectomy at the time of aortic valve replacement (AVR) only when there is dynamic obstruction with SAM of the mitral valve. Whereas Turina^[5]^ has advised more liberal use of septal myectomy during AVR. During the first surgery, no obstruction was found indicating for myectomy. However, in this case, we believe the thickened septum somewhat prevented the St Jude Medical Regent prosthesis from working normally. Myocardial edema after surgery was one of the promoting factors. In this situation, a Konno operation may be considered. However, we recommend concomitant septal myectomy for patients with hypertrophy septum or a little rotation of pivot in St Jude Medical Regent prosthesis replacement surgery to lower the risk of valve dysfunction.

## Supplementary Material

Supplemental Digital Content
